# LINE FUSION GENES: a database of LINE expression in human genes

**DOI:** 10.1186/1471-2164-7-139

**Published:** 2006-06-07

**Authors:** Dae-Soo Kim, Tae-Hyung Kim, Jae-Won Huh, Il-Chul Kim, Seok-Won Kim, Hong-Seog Park, Heui-Soo Kim

**Affiliations:** 1 PBBRC, Interdisciplinary Research Program of Bioinformatics, Pusan National University, Busan 609-735, Korea; 2 Division of Biological Sciences, College of Natural Sciences, Pusan National University, Busan 609-735, Korea; 3 National Genome Information Center, Korea Research Institute of Bioscience and Biotechnology, 52 Oun-dong, Yuson-gu, Daejeon 305-333, Korea

## Abstract

**Background:**

Long Interspersed Nuclear Elements (LINEs) are the most abundant retrotransposons in humans. About 79% of human genes are estimated to contain at least one segment of LINE per transcription unit. Recent studies have shown that LINE elements can affect protein sequences, splicing patterns and expression of human genes.

**Description:**

We have developed a database, LINE FUSION GENES, for elucidating LINE expression throughout the human gene database. We searched the 28,171 genes listed in the NCBI database for LINE elements and analyzed their structures and expression patterns. The results show that the mRNA sequences of 1,329 genes were affected by LINE expression. The LINE expression types were classified on the basis of LINEs in the 5' UTR, exon or 3' UTR sequences of the mRNAs. Our database provides further information, such as the tissue distribution and chromosomal location of the genes, and the domain structure that is changed by LINE integration. We have linked all the accession numbers to the NCBI data bank to provide mRNA sequences for subsequent users.

**Conclusion:**

We believe that our work will interest genome scientists and might help them to gain insight into the implications of LINE expression for human evolution and disease.

**Availability:**

## Background

Most retroelements have been considered harmful because they cause accumulation of insertion and deletion mutations in the host genome [1]. Mutation of retroelements could affect gene transcription and translation. However, recent investigations have shown that HERV and Alu elements in the intron or flanking regions of functional human genes provide alternative promoters, splicing sites and polyadenylation signals [2,3]. Unlike HERV and Alu, LINE elements tend to contain multiple potential splice sites (ESE) [4] and polyadenylation signals [5] in their sequences. There are four types of transposable elements in the human genome: long interspersed nuclear elements (LINEs or L1s) or non-long terminal repeat retrotransposons, short interspersed nuclear elements (SINEs), LTR retrotransposons (endogenous retroviruses) and DNA transposons [1], which together constitute 45% of the total genome. Most of these elements are inactive. However, a few LTR elements have been shown to contain intact open reading frames (ORFs) [6], and LINE elements also have the capacity for autonomous retrotransposition [7,8]. SINE elements cannot be expressed by themselves and depend on L1 elements for active mobility [9]. The L1 elements constitute about 17% of the human genome and are present in an estimated 79% of human genes in at least one copy [10].

The full length of L1 is about 6 kb. It consists of a 5' untranslated region (5'UTR); two nonoverlapping open reading frames (ORF1 and ORF2) encoding an RNA binding protein [11], an endonuclease [12] and a reverse transcriptase [13]; and a 3'UTR that ends in an AATAAA polyadenylation signal and a polyA tail [9]. The Alu and SVA transposable elements and processed pseudogenes are believed to have been inserted into the genome by borrowing the endonuclease and reverse transcriptase from L1 elements [14-16]. The L1 element itself has also been inserted into new genomic locations during mammalian evolution. Such elements are mostly truncated and rearranged to form inactive copies of their progenitors. These insertional mutations are reported to be associated with twelve genetic diseases [17] and also contribute to protein variability or versatility [18].

Active or functional L1 elements, which are involved in shaping the human genome, are differentiated into three types depending on where they are inserted into the genome. First, a 6 kb-long full-length or variable-length 5'-truncated L1 element is inserted into the 5'UTR or introns of a gene, affecting its expression. In this process, LINE elements are probably reverse transcribed and integrated in the new location by target-primed reverse transcription (TPRT) [19]. LINE elements have provided not only many internal promoters at new genomic locations, but also 5'-UTR-located internal promoters, which could guide the transcription of many adjacent genes [20]. Second, retrotransposition of the L1 element results in the transduction of a 3'-UTR flanking fragment to a new genomic location; this is due to the effect of the ambiguous L1 polyadenylation signal [21]. Third, the L1 components are shuffled into exons, affecting the splicing site at transcription and consequently leading to the production of alternative mRNA transcripts [22].

Assembling genomic information and constructing a web-database of genome annotations and genes with particular functions is generally useful for implementing functional studies and for understanding evolutionary genomic organization. Representative web-databases of transposable elements in the human genome have been reported: a database of Alu elements incorporated within protein-coding gene [2], an HERV expression and structure analysis system [3] and a system for extrapolating functional annotation to the prediction of active LINE-1 elements [23]. Although it is well established that information about the structure and position of LINE elements in genes is important for functional studies of genetic diseases, such data are limited and are not included in any database that allows large amounts of scattered information to be searched easily. To address this deficiency, we developed a database for LINE expression and structure in the human genome, LINE FUSION GENES. Our database provides the structures and expression patterns of LINE elements including their relative positions in the genes, and additional information such as the tissue distribution and chromosomal location of the genes and their domain structures. To enhance ease of access for subsequent users, we linked all of the accession numbers to the NCBI data bank to provide mRNA sequences.

## Construction and content

### Identification of transcript variants by LINE insertion (LINE FUSION GENES)

First, 28,171 mRNA human-gene sequences and human expressed sequence tags (EST) were downloaded from the NCBI database Build 35 (INSDC, ) and aligned with genomic assembly sequences (Build 35) using the SIM4 program [24]. Only alignments showing >97% sequence identity were used for further stages. As a result, we extracted positional information about the exon and genome sequences to be matched. On the basis of this information we collected contiguous sequences from 5 kb upstream of the 5'UTR end to the same distance downstream of the 3' UTR end. All the sequences were stored as mapping data for each gene. In addition, the DNA sequences of the LINE elements (LINE-1, LINE-2, LINE-3) were downloaded from Repbase Update [25]. We constructed a LINE component library, using BLASTX, from these 205 downloaded sequences, which included 5'UTR, ORF1, ORF2 and 3'UTR.

We used RepeatMasker  to search for LINE sequences in the contiguous segments. For each gene entry, LINE locations on the contig, orientation and sequence were stored in the database. The locations of LINEs and exons on each contig were calculated from their positions. We then merged them on the basis of their positions and found that 4,489 LINEs were fused on 5' UTR (1,392), 3'UTR (2,167) and exonization (930). Finally, we constructed the LINE FUSION GENES database for chimeric transcripts containing L1-5'UTR heads and cellular sequence tails (102) and L1-3'UTR incorporated within transcripts tails (676), and the LINE elements that led to novel splice variants (632). Information about tissue expression and pathogenic LINE fusion transcripts was obtained by gene expression vocabulary (eVOC) annotations of cDNA library sources [26].

### Classification of the LINE FUSION GENES

**Figure 1:**
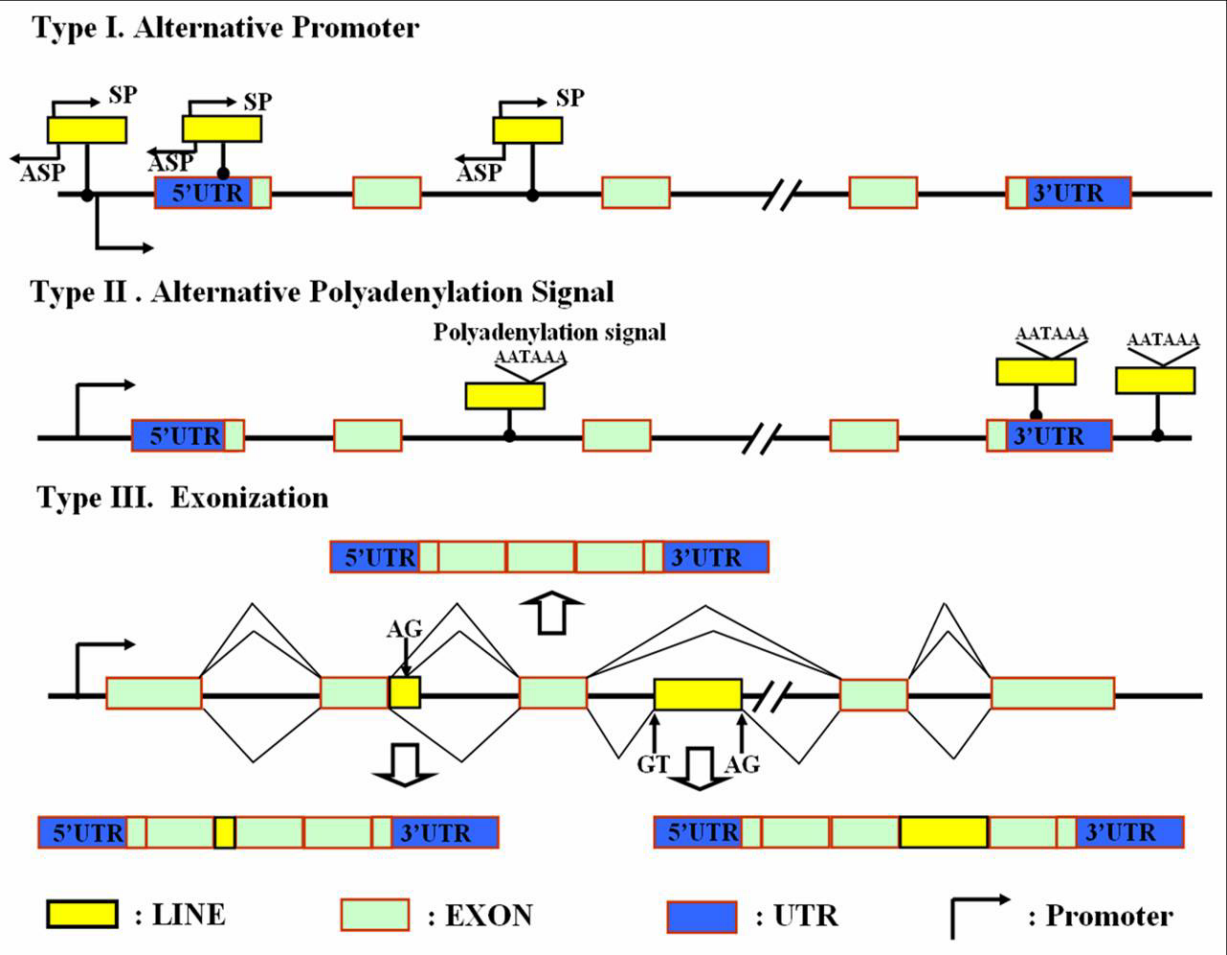
Classification of the LINE fusion types. LINE FUSION GENES were classified into three types. (Type I) Alternative promoter: the promoters of LINEs incorporated near the 5'UTR or into an intron of the gene can act as antisense (ASPs) or sense (SPs) promoters, producing chimeric transcripts different from those of that gene. (Type II) Alternative Poly A signal: LINEs with the poly A signal incorporated in the gene can affect the transcription process resulting in alternative transcripts. (Type III) Exonization: LINEs can be recognized as splicing sites (AG-GT) or intact exons by the spliceosome. LINE element is indicated by yellow box, exon by green box and 5'-3'UTR by blue box.

As shown Figure 1, we classified the LINE FUSION GENES into three types, alternative promoter, alternative polyadenylation signal and exonization, on the basis of the effects of their insertion in the genes. These effects of LINE insertion depend on position and sequence.

#### Type I. Alternative promoter

LINE FUSION GENES of Type I involve insertion near the 5'UTR of the gene or in an intron. LINEs have their own sense and antisense promoters in their 5'UTRs. Consequently, Type I genes might be transcribed from the promoters of the inserted LINE rather than from the cellular promoter. Previously, several cases of Type I LINE FUSION GENES have been reported [27].

#### Type II. Alternative polyadenylation signal

If LINE elements have a polyadenylation signal within the 3' UTR gene flanking region, they could be responsible for a transduction event [8]. Such LINE expression occurs occasionally in human genes; the transcript is stopped by the LINE polyadenylation signal rather than the one endogenous to the gene. When the LINE is incorporated into the intron behind the 3'UTR, transcription is again occasionally stopped by the LINE polyadenylation signal rather than that of the gene. We classified such genes as Type II LINE FUSION GENES. In other words, Type II LINE FUSION GENES are LINE fusion genes with LINE polyadenylation signals on their 3' UTRs.

#### Type III. Exonization

Generally, the intron sequences are spliced out by the spliceosome, which recognizes the splicing site (AG-GT) between the intron and the exon. Most LINEs inserted into introns are spliced out and do not affect target gene expression. However, recent studies have shown that some LINEs can be recognized as splicing sites (AG-GT) or as intact exons by the spliceosome [28]. Consequently, the LINE sequences are fused to mRNA coding sequences. We classified these genes as Type III LINE FUSION GENES.

## Utility and discussion

LINE FUSION GENES uses JSP technology; the data come from a primary database. Users can efficiently retrieve three modes of information concerning LINE expression within genes. First, they can search LINE expression within a gene by typing a gene ID or clicking on the gene name listed on the view page according to its chromosomal location. Second, the database provides type information in which LINE expression is classified into three types (alternative promoter, alternative polyadenylation signal and exonization). The type information can help users to speculate more readily about the effects of LINE expression within interesting genes. Third, users can search interesting genes using accession numbers from the NCBI data bank or from the HUGO symbol name provided on the view page, and even acquire mRNA sequences from the NCBI data bank for further study.

**Figure 2:**
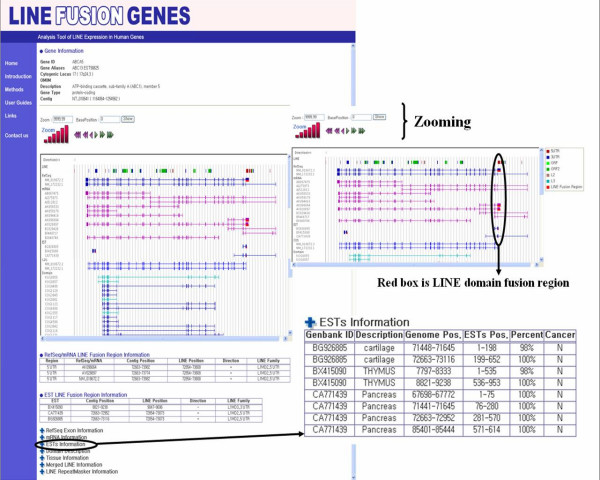
Part of output from LINE FUSION GENES. LINE FUSION GENES shows evidence of and information about expressed LINE events within genes. Both the LINE fusion regions and transcript information are shown in tabular form and a graphic view represents the family, orientation, structure and length of the LINE. This view provides more information such as the tissue distribution of the genes, merging LINE elements as evidence of their expression, and domain information related to LINE expression.

The result pages are listed in a tabular format that provides the evidence for and information about LINE expression within genes. As shown in Figure 2, the LINEs are visualized by colors: red (5' UTR elements), blue (3' UTR elements) and green (ORF1 and ORF2). LINE fusion regions within mRNAs are indicated in red. Moreover, detailed information about the LINE fusion regions are displayed in the table on the result page. Occasionally, LINE incorporation results in domain changes in a protein. In order to speculate about these domain changes, users can check the domain description on the page. The domain information includes the results obtained from searching queries about genes with LINEs by RPS-BLAST [29].

## Conclusion

From our in silico analysis of the human genome, 1,329 genes were identified as being affected by LINE elements during expression. LINE FUSION GENES is continually supplemented with new human gene data from the available sources. We are planning to update the database with full length human cDNA data obtained from various clinical samples representing human diseases. Through this update, we will be able to profile the patterns of LINE expression in various diseases and to identify LINEs that affect the expression of functional human genes. We will also supplement the database with LINE fusion genes from other mammalian species and compare them with those of humans. We also envision the integration of our HESAS [3] and LINE FUSION GENES databases, intended for release in 2007. We believe that our work will help us to gain insight into the implications of LINE expression for human evolution and disease.

## Availability and requirements

LINE FUSION GENES is publicly available at the URL . Questions and comments are welcomed through the site.

## Abbreviations

LINE – Long Interspersed Element

HERV – Human Endogenous Retrovirus

SINE – Short Interspersed Nucleotide Element

ORF – Open Reading Frame

BLAST – Basic Local Alignment Search Tool

JSP – Java Server Pages

RPS-BLAST – Reversed Position Specific Blast

HESAS – HERVs Expression and Structure Analysis System

EST – Expressed Sequence Tag

UTR – Untranslated Regions

NCBI – National Center for Biotechnology Information

HUGO – Human Genome Organisation

INSDC – International Nucleotide Sequence Databases

## Authors' contributions

DS Kim analyzed the contents of the paper and wrote the manuscript. HS Kim participated in the analysis and provided essential direction. TH Kim provided biological context and guidance during the initial phase of the bioinformatics analysis. HS Park and IC Kim contributed the manuscript correction and continuous discussions. SW Kim helped in the general design of the database and the user interface. JW Huh provided biological direction. All authors read and approved the final manuscript.
